# Identification of Stochastically Perturbed Autonomous Systems from Temporal Sequences of Probability Density Functions

**DOI:** 10.1007/s00332-018-9455-0

**Published:** 2018-03-21

**Authors:** Xiaokai Nie, Jingjing Luo, Daniel Coca, Mark Birkin, Jing Chen

**Affiliations:** 1 Leeds Institute for Data Analytics, University of Leeds, Leeds, LS2 9JT UK; 2 Department of Automatic Control and Systems Engineering, The University of Sheffield, Sheffield, S1 3JD UK; 3 Institute of AI and Robotics, Academy for Engineering and Technology, Fudan University, Shanghai, 200433 China; 4 Warwick Medical School, The University of Warwick, Coventry, CV4 7AL UK

**Keywords:** Nonlinear systems, Probability density functions, Frobenius–Perron operator, Stochastic dynamical systems, 93E12, 37H99, 65P40

## Abstract

The paper introduces a method for reconstructing one-dimensional iterated maps that are driven by an external control input and subjected to an additive stochastic perturbation, from sequences of probability density functions that are generated by the stochastic dynamical systems and observed experimentally.

## Introduction

There is considerable interest in modeling and analyzing dynamical systems that generate densities of states. Examples of such systems include chaotic systems (Boyarsky and Góra 1997; Lasota and Mackey 1994) and stochastically perturbed dynamical systems (Swishchuk and Islam 2013). Such systems are encountered routinely in physics, biology, engineering and economics (Strogatz 2014; Skinner 1994).

In many practical situations, the system that generates the density of states is unknown and only the densities of states generated by the system or the invariant density associated with the system can be observed, while the individual point trajectories are not measurable. Conventional solutions (Maguire et al. 1998; Han et al. 2004; Príncipe and Kuo 1995; Lai et al. 1999; Lai and Tél 2011; Bollt et al. 2001) rely on time series observations, but for such situations they become unsuitable. The problem of inferring the unknown dynamical system from the observed densities is known as the inverse Frobenius–Perron problem (Boyarsky and Góra 1997; Ershov and Malinetskii 1988). The problem of reconstructing an unknown one-dimensional autonomous chaotic map given only knowledge of the invariant density function of the system has been considered by a number of authors (Ershov and Malinetskii 1988; Góra and Boyarsky 1993; Diakonos and Schmelcher 1996; Pingel et al. 1999), while there are special cases in which this problem has a unique solution. Given the invariant symmetric beta density functions, methods were introduced to construct a class of symmetric maps (Diakonos and Schmelcher 1996) and a broader class of continuous unimodal maps whose each brand covers the complete interval (Pingel et al. 1999). Given arbitrary invariant densities other similar approaches were proposed for identifying the maps with specified forms: two types of one-dimensional symmetric maps (Koga 1991), smooth chaotic map with closed form (Huang 2006, 2009), multi-branches complete chaotic map (Huang 2009). Problems of synthesizing one-dimensional maps with prescribed invariant density function or autocorrelation function were tackled in Baranovsky and Daems (1995) and Diakonos et al. (1999). Using positive matrix theory an approach to synthesizing chaotic maps with arbitrary piecewise constant invariant densities and arbitrary mixing properties was developed in Rogers et al. (2004). This method was further extended to synthesizing dynamical systems with desired statistical properties (Rogers et al. 2008a), developing communication networks (Berman et al. 2004) and designing randomly switched chaotic maps and two-dimensional chaotic maps used for image generation (Rogers et al. 2008b). In Bollt (2000) and Bollt and Santitissadeekorn (2013), a global and open-loop strategy of controlling chaos was presented to solve the inverse problem. The problem was reduced to that of finding a perturbation of the original Frobenius–Perron matrix to achieve the target invariant density function. In general, given only invariant density function, the solution to the inverse problem is not unique, as different maps exhibiting remarkably different dynamics may possess a same invariant density function. Therefore, additional assumptions or constraints are required to ensure the uniqueness of the identification results. A more recent approach (Nie and Coca 2015) addresses the uniqueness issue by considering sequences of density functions generated by the system rather than just the invariant density function of the system. This method allows inferring the map that exhibits the same transient and asymptotic dynamics as the underlying system that generated the data. Although it is shown that the method is robust to noise, the approach does not exploit any a priori knowledge of the noise distribution. In addition, to our knowledge, all existing methods consider only autonomous maps.

In this context, this paper introduces for the first time a method to infer a one-dimensional map that is driven by an external control input while being subjected to an additive stochastic perturbation from sequences of observed density functions generated by the unknown system. We formulate the operator transferring the state density function of the stochastic dynamical system in terms of the Frobenius–Perron operator associated with the unperturbed underlying system that we aim to estimate, and derive the matrix representation of the transfer operator in terms of the Frobenius–Perron matrix. Based on this representation the new algorithm is developed to estimate the Frobenius–Perron matrix using temporal sequences of probability density functions generated by the stochastic dynamical system given the density functions of the control input and noise. The approach also determines the monotonicity of general nonlinear transformations over each interval of the partition, which is a crucial step to reconstruct the true dynamical system.

The paper is structured as follows. Section 2 introduces the inverse problem. The stochastic Frobenius–Perron operator associated with stochastically perturbed autonomous systems is derived in Sect. 3. A matrix approximation of the operator is given in Sect. 4. Section 5 introduces the methodology of reconstructing general nonlinear maps from sequences of density functions. Section 6 presents a numerical simulation example to demonstrate the effectiveness of the developed algorithm for the stochastically perturbed autonomous systems. Conclusions are given in Sect. 7.

## Inverse Problem Formulation

Let (*R* = [0,*b*], ${\mathcal{B}}$, *μ*) be a normalized measure space, where *μ* is a measure on (*R*, ${\mathcal{B}}$) and ${\mathcal{B}}$ is a Borel $\sigma$-algebra of subsets in *R.* Consider the following discrete-time stochastic dynamical system

1 $$ x_{n + 1} = S(x_{n} ) + u_{n} + \xi_{n} \quad (\bmod \,b ) , $$

where $S{:}\,R \to R$ is a measurable and nonsingular transformation [i.e., $\mu (S^{ - 1} (A)) = {\mathcal{B}}$ for any $A \in {\mathcal{B}}$ and if $\mu (S^{ - 1} (A)) = 0$ for all $A \in {\mathcal{B}}$, then $\mu (A) = 0$], $x_{n} \in R$ is the state variable having the probability density function $f_{n} \in D(R,{\mathcal{B}},\mu ),\,D = \{ f \in L^{1} (R,{\mathcal{B}} ,\mu){:}\,f \ge 0,\left\| f \right\|_{1} = 1\}, {\mkern 1mu} u_{n} \in R$ is the control input of the system with a probability density function $f_{u} \in L^{1} (R)$ that can be assigned, and $\xi_{n}$ is an independent random variable with a known probability density function *g* that has compact support on $[ - \,\varepsilon ,\varepsilon ]$, that is, $\xi_{n}$ is bounded in $[ - \,\varepsilon ,\varepsilon ]$, $\varepsilon \le b$.

Let $X_{0,i} = \{ x_{0,j}^{i} \}_{j = 1}^{\theta }$ and $X_{1,i} = \{ x_{1,j}^{i} \}_{j = 1}^{\theta }$, *i* = 1, …, *K* be random vectors of initial and final state observations, respectively, such that

2 $$ x_{1,j}^{i} = S(x_{0,j}^{i} ) + u_{0,j} + \xi_{0,j} \quad (\bmod \;b), $$

where $i = 1, \ldots ,K$. Assuming that for practical reasons it is not possible to track individual point trajectories during the experiment, that is to associate an initial state $x_{0,j}^{i}$ with its image $x_{1,j}^{i}$ under the transformation, the inverse problem considered in this paper is to infer the point transformation *S* in (1) from the probability density functions $f_{0,j}$ and $f_{1,j}$ of the initial and final states $X_{0,i} = \{ x_{0,j} \}_{j = 1}^{\theta }$ and $X_{1,i} = \{ x_{1,j} \}_{j = 1}^{\theta }$, *i* = 1, …, *K*.

## The Stochastic Frobenius–Perron Operator Associated with the Stochastically Perturbed Transformation

In this section the transfer of density function at *n* to *n* + 1 is derived given the input and noise density functions $f_{u}$ and *g*. For a dynamical system with a constantly applied random perturbation written in the following general form (Lasota and Mackey 1994)

3 $$ x_{n + 1} = \bar{S}(x_{n} ,\xi_{n} ) = S(x_{n} ) + \xi_{n} , $$

where $S{:}\,R \to R$ is a given transformation and $\xi_{n}$ is an independent random variable having a density function *g*. The operator transferring state density functions of the perturbed dynamical system are called the stochastic Frobenius–Perron operator, denoted by $\bar{P}$,

4 $$ \bar{P}f(x) = \int_{R} {\tau (x,y)f(y)} {\kern 1pt} {\text{d}}y, $$

where $\tau (x,y) = g(x - S(y))$ is a stochastic kernel, satisfying $\tau (x,y) > 0$, and $\int_{R} {\tau (x,y)} = 1$. For a nonsingular unperturbed transformation *S*, the Frobenius–Perron operator (Boyarsky and Góra 1997) corresponding to *S* exists, denoted by $P_{S}$, and (4) is further written as

5 $$ \bar{P}f(x) = \int_{R} {g(y)P_{S} f(x - y)} {\kern 1pt} {\text{d}}y. $$

Let $G{:}\,R \times R \to R$ be defined by

6 $$ G(x_{n} ,u_{n} ) = S(x_{n} ) + u_{n} \quad ( {\text{mod}}\,b ) , $$

such that (1) can be written as

7 $$ x_{n + 1} = G(x_{n} ,u_{n} ) + \xi_{n} \quad ( {\text{mod}}\,b ). $$

Let $\bar{x}_{n + 1} = G(x_{n} ,u_{n} ) \in R$. From (5) it follows that the probability density function of $\bar{x}_{n + 1}$ is given by

8 $$ \bar{f}_{n + 1} (\bar{x}) = \int_{R} {f_{u} \left[ {\bar{x} - y + b\chi_{R} (y - \bar{x})} \right]P_{S} f_{n} (y){\kern 1pt} {\text{d}}y} . $$

where $\chi_{\varDelta } (x)$ is the indicator function defined by

9 $$ \chi_{\varDelta } (x) = \left\{ {\begin{array}{*{20}l} 1 \hfill & {{\text{if}}\,x \in \varDelta ;} \hfill \\ 0 \hfill & {{\text{if}}\;x \notin \varDelta .} \hfill \\ \end{array} } \right. $$

Equation (7) becomes

10 $$ x_{n + 1} = \bar{x}_{n + 1} + \xi_{n} \quad ( {\text{mod}}\,b ) , $$

where the probability density function of $x_{n + 1}$ is given by

11 $$ f_{n + 1} (x) = \int_{R} {\bar{f}_{n + 1} (\bar{x})g\left[ {x - \bar{x} + b\chi_{( - b,\varepsilon - b]} (x - \bar{x}) - b\chi_{[b - \varepsilon ,b)} (x - \bar{x})} \right]} {\text{d}}\bar{x}, $$

Substituting (8) into (11) leads to the following formulation of the stochastic Frobenius–Perron operator, denoted by $\bar{P}$, associated with stochastic dynamical system (1)

12 $$ \begin{aligned} \bar{P}f_{n} (x) & = f_{n + 1} (x) = \int_{R} {\int_{R} {f_{u} [\bar{x} - y + b\chi_{R} (y - \bar{x})]} } \\ & \quad \cdot g\left[ {x - \bar{x} + b\chi_{( - b,\varepsilon - b]} (x - \bar{x}) - b\chi_{[b - \varepsilon ,b)} (x - \bar{x})} \right]P_{S} f_{n} (y){\kern 1pt} {\text{d}}y{\text{d}}\bar{x}. \\ \end{aligned} $$

Equation (12) relates the operator $\bar{P}$, corresponding to the stochastic system, to the Frobenius–Perron operator $P_{S}$ associated with the map *S*. This equation forms the basis for the new approach to reconstruct the map *S* based on sequences of density functions.

### *Remark 1*

The additive noise $\xi_{n}$ is an i.i.d. random variable that normally satisfies that $\hbox{max} (|\xi_{n} |) < b$ in general practical measurements. For the unusual case $\varepsilon > b,$ (10) can be rewritten as

13 $$ x_{n + 1} = \bar{x}_{n + 1} + \xi_{n} - k^{1} b\chi_{(b, + \infty )} (\bar{x}_{n + 1} + \xi_{n} ) + k^{2} \chi_{( - \infty ,0)} (\bar{x}_{n + 1} + \xi_{n} ), $$

where $k^{1} = \left\lfloor {\frac{{\bar{x}_{n + 1} + \xi_{n} }}{b}} \right\rfloor ,\,k^{2} = \left| {\left\lfloor {\frac{{\bar{x}_{n + 1} + \xi_{n} }}{b}} \right\rfloor } \right|$. Since $k^{1}$ and $k^{2}$ have infinite results given only $x$ and $\bar{x}$, $f_{n + 1}$ cannot be uniquely generated from $\bar{f}_{n + 1}$ in (11). Hence, $\xi_{n}$ is treated as a variable bounded in $[ - \,\varepsilon ,\varepsilon ]$, $\varepsilon \le b$.

### *Remark 2*

An alternative compact way of formulating the stochastic Frobenius–Perron operator is to apply the joint density function denoted by $f_{\alpha } \in L^{1} (R)$ for the control input and noise to (4). Let $\alpha_{n} = u_{n} + \xi_{n}$ (mod *b*). Thus, $f_{\alpha }$ can be given in terms of $f_{u}$ and *g* by

14 $$ f_{\alpha } (\alpha ) = \int_{R} {g\left[ {\alpha - u + b\chi_{( - b,\varepsilon - b]} (\alpha - u) - b\chi_{[b - \varepsilon ,b)} (\alpha - u)} \right]} f_{u} (u){\text{d}}u. $$

It follows that $x_{n + 1} = S(x_{n} ) + \alpha_{n}$ (mod *b*), and from (5) we have that

15 $$ f_{n + 1} (x) = \int_{R} {f_{\alpha } (z)P_{S} f_{n} (x - z + b\chi_{R} (z - x))} {\text{d}}z. $$

Substituting (14) into (15) gives that

16 $$ \begin{aligned} f_{n + 1} (x) & = \int_{R} {\int_{R} {g[z - u + b\chi_{( - b,\varepsilon - b]} (z - u) - b\chi_{[b - \varepsilon ,b)} (z - u)]} } f_{u} (u) \\ & \quad P_{S} f_{n} (x - z + b\chi_{R} (z - x)){\text{d}}z{\text{d}}u \\ & = \int_{R} {\int_{R} {g[x - y + b\chi_{R} (y - x) - u + b\chi_{( - b,\varepsilon - b]} (x - y + b\chi_{R} (y - x) - u)} } \\ & \quad - b\chi_{[b - \varepsilon ,b)} (x - y + b\chi_{R} (y - x) - u)]f_{u} (u)P_{S} f_{n} (y){\text{d}}y{\text{d}}u \\ \end{aligned} $$

Let $x - \bar{x} = x - y + b\chi_{R} (y - x) - u$, then $u = \bar{x} - y + b\chi_{R} (y - \bar{x})$. It follows that (12) is obtained from (16).

In the first instance, we assume that *S* belongs to a special class of nonlinear transformations called piecewise linear semi-Markov transformations and develop the algorithm to reconstruct it. We then show how the reconstruction approach can be applied to approximate more general one-dimensional maps.

## A Matrix Representation of the Transfer Operator $\bar{P}$

Let *S* be a piecewise linear and expanding semi-Markov transformation over the *N*-interval partition, $\Re = \{ R_{1} ,R_{2} , \ldots ,R_{N} \} .$

### **Definition 1**

A transformation $S{:}\,R \to R$ is said to be *semi*-*Markov* with respect to the partition $\Re$ (or $\Re$-semi-Markov) if there exist disjoint intervals $Q_{j}^{(i)}$ so that $R_{i} = \cup_{k = 1}^{p(i)} Q_{k}^{(i)}$, $i = 1, \ldots ,N$, the restriction of *S* to $Q_{k}^{(i)}$, denoted $\left. S \right|_{{Q_{k}^{(i)} }}$, is monotonic and $S(Q_{k}^{(i)} ) \in \Re .$ (Góra and Boyarsky 1993)

The restriction $\left. S \right|_{{R_{i} }}$ is a homeomorphism from $R_{i}$ to a union of intervals of $\Re$

17 $$ \bigcup\limits_{k = 1}^{p(i)} {R_{r(i,k)} } = \bigcup\limits_{k = 1}^{p(i)} {S(Q_{k}^{(i)} )} , $$

where $R_{r(i,k)} = S(Q_{k}^{(i)} ) \in \Re ,\,Q_{k}^{(i)} = [q_{k - 1}^{(i)} ,q_{k}^{(i)} ],\,i = 1, \ldots ,N,\,k = 1, \ldots ,p(i)$ and $p(i)$ denotes the number of disjoint subintervals $Q_{k}^{(i)}$ corresponding to $R_{i}$.

Let $f_{n}$ be a piecewise constant function over the partition $\Re$ such that $f_{n} (x) = \sum\nolimits_{i = 1}^{N} {w_{i}^{n} \chi_{{R_{i} }} (x)}$. According to the property of semi-Markov map (Boyarsky and Góra 1997), its image under transformation $P_{S} f_{n}$ is also a piecewise constant function over $\Re$ such that $P_{S} f_{n} (x) = \sum\nolimits_{i = 1}^{N} {\varphi_{i}^{n} \chi_{{R_{i} }} (x)}$. In this case, the Frobenius–Perron operator can be represented by a finite-dimensional matrix such that

18 $$ P_{S} f_{n} (x) = \sum\limits_{j = 1}^{N} {\left( {\sum\limits_{i = 1}^{N} {(w_{i}^{n} m_{i,j} )} } \right)} \chi_{{R_{j} }} (x), $$

where $M = (m_{i,j} )_{1 \le i,j \le N}$ is the Frobenius–Perron matrix induced by *S* with entries given by

19 $$ m_{i,j} = \left\{ {\begin{array}{*{20}l} {\left| {\left. {(S} \right|_{{Q_{j}^{(i)} }} )^{{\prime }} } \right|^{ - 1} ,} \hfill & {{\text{if}}\,S(Q_{k}^{(i)} ) = R_{j} ;} \hfill \\ {0,} \hfill & {{\text{otherwise}}.} \hfill \\ \end{array} } \right. $$

From (18) it follows that

20 $$ \varphi_{j}^{n} = \sum\limits_{i = 1}^{N} {w_{i}^{n} m{}_{i,j}} , $$

for $j = 1, \ldots ,N$. Let $w^{{f_{n}^{N} }} = [w_{1}^{n} ,w_{2}^{n} , \ldots ,w_{N}^{n} ],\,\varphi^{{P_{S} f_{n}^{N} }} = [\varphi_{1}^{n} ,\varphi_{2}^{n} , \ldots ,\varphi_{N}^{n} ]$ be the coefficient vectors of the piecewise constant density functions $f_{n}$ and $P_{S} f_{n}$ over the partition $\Re$, respectively. We have $\varphi^{{P_{S} f_{n}^{N} }} = w^{{f_{n}^{N} }} M$.

By integrating both sides of (12) over $R_{i} \in \Re ,$ it follows that

21 $$ \begin{aligned} \int_{{R_{i} }} {\bar{P}f_{n} (x){\text{d}}x} & = \int_{{R_{i} }} {\int_{R} {\int_{R} {f_{u} [\bar{x} - y + b\chi_{R} (y - \bar{x})]} } } \\ & \quad \cdot g[x - \bar{x} + b\chi_{( - b,\varepsilon - b]} (x - \bar{x}) - b\chi_{[b - \varepsilon ,b)} (x - \bar{x})]P_{S} f_{n} (y){\kern 1pt} {\text{d}}y{\text{d}}\bar{x}{\text{d}}x. \\ \end{aligned} $$

For $f_{n} \in L^{1}$ we define

22 $$ f_{n + 1}^{N} = \bar{P}_{N} f_{n} (x) = \sum\limits_{i = 1}^{N} {w_{i}^{n + 1} \chi_{{R_{i} }} (x)} , $$

where

23 $$ \begin{aligned} w_{i}^{n + 1} & = \frac{1}{{\lambda (R_{i} )}}\int_{{R_{i} }} {\int_{R} {\int_{R} {f_{u} [\bar{x} - y + b\chi_{R} (y - \bar{x})]} } } \\ & \quad \cdot g\left[ {x - \bar{x} + b\chi_{( - b,\varepsilon - b]} (x - \bar{x}) - b\chi_{[b - \varepsilon ,b)} (x - \bar{x})} \right]P_{S} f_{n} (y){\kern 1pt} {\text{d}}y{\text{d}}{\kern 1pt} \bar{x}{\text{d}}x, \\ \end{aligned} $$

$\lambda$ denotes the Lebesgue measure and $f_{n + 1}^{N}$ denotes the piecewise constant approximation of $f_{n + 1}$ over the partition $\Re$. We have the following result (Li 1976).

### **Lemma 1**

*For* $f \in L^{1}$, *the sequence* $\bar{P}_{N} f(x) = \sum\nolimits_{i = 1}^{N} {w_{i} \chi_{{R_{i} }} (x)}$ *converges in* $L^{1}$ *to* $\bar{P}f$ *as* $N \to + \infty$.

Substituting (17) in (21) gives

24 $$ \begin{aligned} w_{i}^{n + 1} & = \frac{1}{{\lambda (R_{i} )}}\int_{{R_{i} }} {\sum\limits_{j = 1}^{N} {\int_{{R_{j} }} {\int_{R} {f_{u} [\bar{x} - y + b\chi_{R} (y - \bar{x})]} } } } \\ & \quad \cdot g[x - \bar{x} + b\chi_{( - b,\varepsilon - b]} (x - \bar{x}) - b\chi_{[b - \varepsilon ,b)} (x - \bar{x})]\varphi_{j}^{n} {\text{d}}y{\text{d}}\bar{x}{\text{d}}x \\ & = \frac{1}{{\lambda (R_{i} )}}\sum\limits_{j = 1}^{N} \left\{ \int_{{R_{i} }} \int_{{R_{j} }} \int_{R} f_{u} [\bar{x} - y + b\chi_{R} (y - \bar{x})] \right.\\&\left. \cdot g[x - \bar{x} + b\chi_{( - b,\varepsilon - b]} (x - \bar{x}) - b\chi_{[b - \varepsilon ,b)} (x - \bar{x})]{\kern 1pt} {\text{d}}y{\text{d}}\bar{x}{\text{d}}x \cdot \varphi_{j}^{n} \right\} . \\ \end{aligned} $$

Let $H = (h_{i,\;j} )_{1 \le i,\;j \le N}$ be a matrix with entries given by

25 $$ \begin{aligned} h_{i,j} & = \frac{1}{{\lambda (R_{i} )}}\sum\limits_{j = 1}^{N} {\left\{ {\int_{{R_{i} }} {\int_{{R_{j} }} {\int_{R} {f_{u} [\bar{x} - y + b\chi_{R} (y - \bar{x})]} } } } \right.} \\ & \quad \left. { \cdot g[x - \bar{x} + b\chi_{( - b,\varepsilon - b]} (x - \bar{x}) - b\chi_{[b - \varepsilon ,b)} (x - \bar{x})]{\text{d}}y{\text{d}}{\kern 1pt} \bar{x}{\text{d}}x} \right\}. \\ \end{aligned} $$

It follows from (20) and (24) that

26 $$ w^{{f_{n + 1}^{N} }} = \varphi^{{P_{S} f_{n}^{N} }} \cdot H^{{\prime }} = w^{{f_{n}^{N} }} \cdot M \cdot H^{{\prime }} . $$

Let $Q = MH^{{\prime }}$. The evolution of density functions is formulated as $w^{{f_{n + 1}^{N} }} = w^{{f_{n}^{N} }} Q$. *Q* is the matrix representation of the transfer operator $\bar{P}$. Formula (26) yields the final density function estimated over the N-interval partition $\Re$, mapping from the initial piecewise constant density function over the N-interval partition $\Re$. This establishes the basis of the new algorithm of reconstructing the unknown transformation S from sequences of probability density functions.

### *Remark 3*

Given the nonsingular transformation $S{:}\,R \to R$ that induces the Frobenius–Perron matrix *M* with respect to the partition $\Re$, input density function $f_{u} \in L^{1}$ and noise density function $g \in L^{1}$, from (26) the estimated state density function over $\Re$ of stochastic dynamical system (1) can be predicted from a piecewise constant initial density function $f_{0}^{N}$ as $w^{{f_{n}^{N} }} = w^{{f_{0}^{N} }} Q^{n} .$

### *Remark 4*

Let $Q = (q_{i,j} )_{1 \le i,j \le N}$, where from (26) $q_{i,j}$ is given by

27 $$ q_{i,j} = \sum\limits_{k = 1}^{N} {(m_{i,k} h_{j,k} )} . $$

Then we have

28 $$ \begin{aligned} \sum\limits_{j = 1}^{N} {q_{i,j} } & = \sum\limits_{j = 1}^{N} {\left( {\left[ {\begin{array}{*{20}c} {m_{i,1} } & \ldots & {m_{i,k} } & \ldots & {m_{i,N} } \\ \end{array} } \right]{\kern 1pt} {\kern 1pt} \left[ {\begin{array}{*{20}c} {h_{j,1} } & \ldots & {h_{j,k} } & \ldots & {h_{j,N} } \\ \end{array} } \right]^{{\prime }} } \right)} \\ & = \left[ {\begin{array}{*{20}c} {m_{i,1} } & \ldots & {m_{i,k} } & \ldots & {m_{i,N} } \\ \end{array} } \right]{\kern 1pt} {\kern 1pt} \left[ {\begin{array}{*{20}c} {\sum\limits_{j = 1}^{N} {h_{j,1} } } & \ldots & {\sum\limits_{j = 1}^{N} {h_{j,k} } } & \ldots & {\sum\limits_{j = 1}^{N} {h_{j,N} } } \\ \end{array} } \right]^{{\prime }} . \\ \end{aligned} $$

It is obtained from (25) that

29 $$ \begin{aligned} \sum\limits_{j = 1}^{N} {h_{j,k} } & = \sum\limits_{j = 1}^{N} {\left( {\frac{1}{{\lambda (R_{j} )}}\sum\limits_{k = 1}^{N} {\left\{ {\int_{{R_{j} }} {\int_{{R_{k} }} {\int_{R} {f_{u} [\bar{x} - y + b\chi_{R} (y - \bar{x})]} } } } \right.} } \right.} \\ & \quad \left. {\left. { \cdot g[x - \bar{x} + b\chi_{( - b,\varepsilon - b]} (x - \bar{x}) - b\chi_{[b - \varepsilon ,b)} (x - \bar{x})]{\kern 1pt} {\text{d}}y{\text{d}}{\kern 1pt} \bar{x}{\text{d}}x} \right\}} \right) \\ & = \frac{N}{b}\int_{R} {\int_{{R_{k} }} {\int_{R} {\left\{ {f_{u} [\bar{x} - y + b\chi_{R} (y - \bar{x})]} \right.} } } \\ & \quad \cdot g[x - \bar{x} + b\chi_{( - b,\varepsilon - b]} (x - \bar{x}) - b\chi_{[b - \varepsilon ,b)} (x - \bar{x})]{\kern 1pt} {\text{d}}y{\text{d}}\bar{x}{\text{d}}x \\ & = 1. \\ \end{aligned} $$

It follows that

30 $$ \sum\limits_{j = 1}^{N} {q_{i,j} } = \sum\limits_{k = 1}^{N} {m_{i,k} } = 1. $$

This implies that matrix *Q* is a stochastic matrix that has 1 as the eigenvalue of maximum modulus, of which the algebraic and geometric multiplicities are 1. Since *Q* and $Q^{{\prime }}$ have the same eigenvalues, we then have $Q^{{\prime }} (w^{{f_{ * }^{N} }} )^{{\prime }} = (w^{{f_{ * }^{N} }} )^{{\prime }}$, thereby $w^{{f_{ * }^{N} }} Q = w^{{f_{ * }^{N} }}$, where $w^{{f_{ * }^{N} }} = [w_{1}^{ * } ,w_{2}^{ * } , \ldots ,w_{N}^{ * } ]$ represents the equilibrium density vector of *Q*.

### *Remark 5*

Remark 4 suggests that there exists a stationary density function $f_{ * }^{N} (x) = \sum\nolimits_{i = 1}^{N} {w_{i}^{ * } \chi_{{R_{i} }} (x)}$ for the transfer operator $\bar{P}_{N}$. It follows from Lemma 1 that $f_{ * }^{N} (x)$ converges to $f_{ * }^{{}} (x)$ of the stochastic dynamical system as $N \to + \infty$.

## Solving the Stochastic Inverse Frobenius–Perron Problem for Continuous Nonlinear Transformations

This section introduces a method to reconstruct the underlying map *S* in Eq. (1) based on a sequence of probability density functions estimated from data, under the general assumption that *S* is a continuous nonlinear map. Specifically, the method infers a piecewise linear semi-Markov map *Ŝ* with respect to a uniform partition $\Re = \{ R_{1} ,R_{2} , \ldots ,R_{N} \} = \{ [0,a_{1} ],(a_{1} ,a_{2} ], \ldots ,\;(a_{N - 1} ,a_{N} ]\} ,\,a_{N} = b$, given *K* random vectors of initial states $X_{0,i} = \{ x_{j}^{0,i} \}_{j = 1}^{\theta }$, from *K* initial state densities $f_{0,i}$, *i* = 1, …, *K*, the corresponding final state vectors $X_{1,i} = \{ x_{j}^{1,i} \}_{j = 1}^{\theta }$, *i* = 1, …, *K* under transformation (1) and the density of the noise and of the control input, *g* and $f_{u}$, respectively. The matrix *M* associated with $P_{S}$ can be approximated arbitrarily well, and thus, *Ŝ* approximates *S* to an arbitrary accuracy as $N \to + \infty$. While *g* is fixed, $f_{u}$ can be defined by the user when the experiment is conducted. It is assumed that the correspondence between an initial state measurement $x_{0,j}$ and its image $x_{1,j}$ under the transformation is not known and hence the point transformation *S* in (1) has to be inferred based on the probability density functions $\left\{ {f_{0,j} } \right\}_{j = 1}^{K} ,\,\left\{ {f_{1,j} } \right\}_{j = 1}^{K}$, *g* and $f_{u}$.

The proposed reconstruction algorithm for general nonlinear and continuous maps is summarized below. However, it is worth emphasizing that this method can also be used in cases when *S* is piecewise semi-Markov.

- *Step 1*: For *K* initial, piecewise constant densities $f_{0,i}$ generate $X_{0,i} = \{ x_{j}^{0,i} \}_{j = 1}^{\theta }$ and $X_{t,i} = \{ x_{j}^{t,i} \}_{j = 1}^{\theta }$, *i* = 1, …, *K, t* = 1, …, *T*.
- *Step 2*: Estimate the coefficient vectors $w^{{f_{t,i}^{N} }} = [w_{1}^{t,i} ,w_{2}^{t,i} , \ldots ,w_{N}^{t,i} ]$ corresponding to the piecewise constant density functions $f_{t,i}^{N} (x)$ that approximate the new state density functions $f_{t,i} (x)$ over the regular partition $\Re$. Compute the matrix *H*;
- *Step 3*: Identify a trial Frobenius–Perron matrix $\hat{M}$ firstly to determine the indices of consecutive positive entries of the matrix *M* that represents the Frobenius–Perron operator $P_{S}$ associated with the optimal approximate map $\hat{S}$ and subsequently a refined matrix *M*;
- *Step 4*: Construct the approximate piecewise linear semi-Markov transformation on $\Re$, and smooth it to obtain the continuous nonlinear map.

These steps are described below in more detail.

### Step 1: Observe Sets of States to Assemble Sequences of Densities

Let $f_{0,i}$ be a set of different initial density functions that is piecewise constant on the partition $\Re$

31 $$ f_{0,i} (x) = \sum\limits_{j = 1}^{N} {w_{j}^{0,i} \chi_{{R_{j} }} (x)} , $$

where the coefficients satisfy $\sum\nolimits_{j = 1}^{N} {w_{j}^{0,i} } = \frac{N}{b}$, *i* = 1, …, *K*.

Let $X_{0,i} = \{ x_{j}^{0,i} \}_{j = 1}^{\theta }$ be the set of initial conditions obtained by sampling $f_{0,i} (x)$, and $X_{t,i} = \{ x_{j}^{t,i} \}_{j = 1}^{\theta }$ be the set of states obtained by applying *t* times Eq. (1) such that $x_{j}^{t,i} = S^{t} (x_{j}^{0,i} ) + u_{i} + \xi_{i}$ (mod *b*) for some $x_{j}^{0,i}$, where $U = \{ u_{i} \}_{i = 1}^{\theta } ,\,\varXi = \{ \xi_{i} \}_{i = 1}^{\theta }$ are generated by sampling $f_{u}$ and *g,* respectively.

From Remark 4, given the input and noise density functions, the generated densities converge to a stationary density function regardless of the initial conditions. Therefore, finite densities characterizing the transient dynamics and evolving from an initial density function can be observed. For *K* sequences of densities, the most dynamical behavior exhibited by the perturbed underlying system can be observed from $T_{m}$ iterations given by $T_{m} = \hbox{min} \{ t_{m} \}$, representing the minimum steps taken to approach the stationary density, where $t_{m}$ is an integral set given by

32 $$ \begin{aligned} t_{m} & = \mathop {\arg \;\hbox{min} }\limits_{t \ge 1} J(t) \\ & = \mathop {\arg \;\hbox{min} }\limits_{t \ge 1} \left( {\sum\limits_{k = 1}^{K} {\sqrt {\int_{R} {(f_{t,k} (x) - f_{t - 1,k} (x))^{2} {\text{d}}x} } } } \right). \\ \end{aligned} $$

Thus, $1 \le T \le T_{m}$. Typically, the interval number of $\Re$ is set by $1 < N \le KT$.

### Step 2: Estimate the Coefficients *w* and Compute the Matrix *H*

The piecewise constant density function $f_{1,i}^{N} (x)$ on the partition $\Re$ is given by

33 $$ f_{1,i}^{N} (x) = \sum\limits_{j = 1}^{N} {w_{j}^{1,i} \chi_{{R_{i} }} (x)} ,\quad w_{j}^{1,i} = \frac{N}{\theta b}\sum\limits_{k = 1}^{\theta } {\chi_{{R_{j} }} (x_{1,k} )} $$

These following matrices are then derived.34and35


Given the input and noise density functions $f_{u}$ and *g*, the matrix *H* is computed from (25).

### Step 3: Identify the Frobenius–Perron Matrix *M*

For a continuous nonlinear map, the corresponding Frobenius–Perron matrix *M* must satisfy that the positive entries in each row are contiguous. Without enough constraints to optimize the matrix, it is generally difficult to identify a very fine matrix straightforwardly. Therefore, initially, a trail Frobenius–Perron matrix is derived to determine the indices of contiguous positive entries in each row, which are then used to refine the matrix. This is carried out in two stages. In the first instance, given (21) the coordinate vectors $\varphi^{{P_{S} f_{n} }}$, $n = 0,\; \ldots ,T - 1,$ are obtained by solving the following constrained optimization problem

36 $$ \mathop {\hbox{min} }\limits_{{0 \le \{ \varphi_{j}^{n} \}_{j = 1, \ldots ,N}^{n = 0, \ldots ,T - 1} \le {N \mathord{\left/ {\vphantom {N b}} \right. \kern-0pt} b}}} \left\| {W^{1} - \varPhi \cdot H^{{\prime }} } \right\|_{F} , $$

subject to $\sum\nolimits_{j = 1}^{N} {\varphi_{j}^{n} } = \frac{N}{b},\,{\text{for}}\,n = 0, \ldots ,T - 1$, where37and $|| \cdot ||_{F}$ denotes the Frobenius norm.

Subsequently, the trial matrix denoted by $\hat{M} = (\hat{m}_{i,j} )_{1 \le i,j \le N}$ is obtained as a solution to the following constrained optimization problem

38 $$ \mathop {\hbox{min} }\limits_{{0 \le \{ \hat{m}_{i,j} \}_{i,j = 1}^{N} \le 1}} \left\| {\varPhi - W^{0} \hat{M}} \right\|_{F} , $$

subject to $\sum\nolimits_{j = 1}^{N} {\hat{m}_{i,j} } = 1$, for $i = 1, \ldots ,N$.

Let ${\mathfrak{P}}^{i} = \{ \hat{r}_{s}^{i} ,\hat{r}_{s}^{i} + 1, \ldots ,\hat{r}_{e}^{i} \}$ be the set of column indices of consecutive positive entries in the *i*th row of $\hat{M}$ and $\hat{r}_{m}^{i} \in {\mathfrak{P}}^{i}$ given by $\hat{m}_{{i,\hat{r}_{m}^{i} }} = \hbox{max} \{ \hat{m}_{i,j} \}_{j = 1}^{N}$. Let $\mathop \cup \nolimits_{k = 1}^{{\hat{p}(i)}} R_{{\hat{r}(i,k)}}$ be a connected union of intervals of $\Re$, which are the images of some connected subintervals $\hat{Q}_{k}^{(i)} ,\,k = 1, \ldots ,\hat{p}(i)$, that is, $R_{{\hat{r}(i,k)}} = S(\hat{Q}_{k}^{(i)} ) \in \Re ,i = 1, \ldots ,N,\hat{p}(i) = \hat{r}_{e}^{i} - \hat{r}_{s}^{i} + 1$ and $\hat{r}(i,k) \in {\mathfrak{B}}^{i}$ are the column indices of the positive entries in the *i*th row of *M* satisfying

39 $$ \hat{r}(i,k + 1) = \hat{r}(i,k) + 1, $$

for $$ i = 1, \ldots ,N,\,k = 1, \ldots ,\hat{p}(i) - 1 $$.

The approximation to the continuous map may have an infinite number of pieces of monotonicity, and each piece $\left. S \right|_{{R_{i} }}$ can be linearly approximated. Thus, for a piecewise linear semi-Markov approximation $\hat{S}$, the maximum and minimum column indices of positive entries on two contiguous rows of *M* are further refined by $r(i,p(i)) = \left\lfloor {{{[\hat{r}(i,\hat{p}(i)) + \hat{r}(i + 1,1)]} \mathord{\left/ {\vphantom {{[\hat{r}(i,\hat{p}(i)) + \hat{r}(i + 1,1)]} 2}} \right. \kern-0pt} 2}} \right\rfloor ,\,r(i + 1,1) = \left\lceil {{{[\hat{r}(i,\hat{p}(i)) + \hat{r}(i + 1,1)]} \mathord{\left/ {\vphantom {{[\hat{r}(i,\hat{p}(i)) + \hat{r}(i + 1,1)]} 2}} \right. \kern-0pt} 2}} \right\rceil$, and $\left. {S^{{\prime }} } \right|_{{Q_{p(i)}^{(i)} }} = \left. {S^{{\prime }} } \right|_{{Q_{1}^{(i + 1)} }}$ if $\frac{1}{{\hat{p}(i + 1)}}\sum\nolimits_{k = 1}^{{\hat{p}(i + 1)}} {\hat{r}(i + 1,k)} > \frac{1}{{\hat{p}(i)}}\sum\nolimits_{k = 1}^{{\hat{p}(i)}} {\hat{r}(i,k)}$ and $\left| {\hat{r}(i + 1,1) - \hat{r}(i,\hat{p}(i))} \right| > 1$; $r(i,1) = \left\lceil {{{[\hat{r}(i,1) + \hat{r}(i + 1,\hat{p}(i + 1))]} \mathord{\left/ {\vphantom {{[\hat{r}(i,1) + \hat{r}(i + 1,\hat{p}(i + 1))]} 2}} \right. \kern-0pt} 2}} \right\rceil$, $r(i + 1,p(i + 1)) = \left\lfloor {{{[\hat{r}(i,1) + \hat{r}(i + 1,\hat{p}(i + 1))]} \mathord{\left/ {\vphantom {{[\hat{r}(i,1) + \hat{r}(i + 1,\hat{p}(i + 1))]} 2}} \right. \kern-0pt} 2}} \right\rfloor$ and $\left. {S^{{\prime }} } \right|_{{Q_{1}^{(i)} }} = \left. {S^{{\prime }} } \right|_{{Q_{p(i + 1)}^{(i + 1)} }}$ if $\frac{1}{{\hat{p}(i)}}\sum\nolimits_{k = 1}^{{\hat{p}(i)}} {r(i,k)} > \frac{1}{{\hat{p}(i + 1)}}\sum\nolimits_{k = 1}^{{\hat{p}(i + 1)}} {r(i + 1,k)}$ and $\left| {\hat{r}(i,1) - \hat{r}(i + 1,\hat{p}(i + 1))} \right| > 1$, and that $\left. {S^{{\prime }} } \right|_{{Q_{2}^{(i)} }} = \left. {S^{{\prime }} } \right|_{{Q_{j}^{(i)} }}$ for $j = 3, \ldots ,p(i) - 1$ if $p(i) \ge 4$, where $Q_{k}^{(i)}$ is the newly formed subinterval, and $\{ r(i,1), \ldots ,r(i,p(i))\}$ are the identified column indices of positive entries in the *i*th row of the matrix *M*.

The refined Frobenius–Perron matrix *M* is then obtained by solving the following optimization problem

40 $$ \mathop {\hbox{min} }\limits_{{0 \le \{ m_{i,j} \}_{i,j = 1}^{N} \le 1}} \left\| {\varPhi - W^{0} M} \right\|_{F} , $$

subject to $$ \sum\nolimits_{k = 1}^{p(i)} {m_{i,r(i,1) + k - 1} } = 1 $$ and $$ m_{i,r(i,k)} > 0, $$ for $$ i = 1, \ldots ,N $$ and $$ m_{i,j} = 0, $$ if $$ j \ne r(i,k) $$, for $$ k = 1, \ldots ,p(i) $$.

### Step 4: Construct the Nonlinear Map

This step involves reconstructing the semi-Markov map that corresponds to the identified Frobenius–Perron matrix *M*. For a continuous map, it started with determining the monotonicity of each branch $\left. S \right|_{{Q_{k}^{(i)} }}$. Let $R_{i}^{{\prime }} = [a_{r(i,1) - 1} ,a_{r(i,p(i))} ]$ be the image of the interval $R_{i}$ under the semi-Markov transformation $\hat{S}$ associated with the identified Frobenius–Perron matrix *M*. Denote $a_{r(i,1) - 1}$ as the starting point of $R_{r(i,1)}$ mapped from the subinterval $Q_{1}^{(i)}$, and $a_{r(i,p(i))}$ as the end point of $R_{r(i,p(i))}$, the image of the subinterval $Q_{p(i)}^{(i)}$. Let $\overline{c}_{i}$ be the midpoint of the image $R_{i}^{{\prime }}$. The sign $\gamma (i)$ of $\{ \left. {\hat{S}^{{\prime }} (x)} \right|_{{Q_{k}^{(i)} }} \}_{k = 1}^{p(i)}$ is given by

41 $$ \gamma (i) = \left\{ {\begin{array}{*{20}l} { - 1,} \hfill & {{\text{if}}\, \, \bar{c}_{i} - \bar{c}_{i - 1} < 0;} \hfill \\ {1,} \hfill & {{\text{if}}\, \, \bar{c}_{i} - \bar{c}_{i - 1} \ge 0;} \hfill \\ {\gamma (i - 1),} \hfill & {{\text{if}}\,\bar{c}_{i} = \bar{c}_{i - 1} ,} \hfill \\ \end{array} } \right. $$

for $$ i = 2, \ldots ,N $$ and $$ \gamma (1) = \gamma (2) $$.

Given that the derivative of $\left. S \right|_{{Q_{k}^{(i)} }}$ is ${1 \mathord{\left/ {\vphantom {1 {m_{i,j} }}} \right. \kern-0pt} {m_{i,j} }}$, the end point $q_{k}^{(i)}$ of subinterval $Q_{k}^{(i)}$ within $R_{i}$ is given by

42 $$ q_{k}^{(i)} = \left\{ {\begin{array}{*{20}l} {a_{i - 1} + \frac{b}{N}\sum\limits_{j = 1}^{k} {m_{i,r(i,j)} } ,} \hfill & {{\text{if }}\gamma (i) = + 1;} \hfill \\ {a_{i - 1} + \frac{b}{N}\sum\limits_{j = 1}^{k} {m_{i,r(i,p(i) - k + 1)} ,} } \hfill & {{\text{if }}\gamma (i) = - 1.} \hfill \\ \end{array} } \right. $$

where $k = 1, \ldots ,p(i) - 1$ and $q_{p(i)}^{(i)} = a_{i} ,\,a_{0} = 0$. The piecewise linear semi-Markov transformation $\hat{S}$ on each subinterval $Q_{j}^{(i)}$ is given by

43 $$ \left. {\hat{S}} \right|_{{Q_{j}^{(i)} }} (x) = \left\{ {\begin{array}{*{20}l} {\frac{1}{{m_{i,j} }}(x - q_{k - 1}^{(i)} ) + a_{j - 1} ,} \hfill & {{\text{if}}\,\gamma (i) = + 1;} \hfill \\ { - \frac{1}{{m_{i,j} }}(x - q_{k - 1}^{(i)} ) + a_{j} ,} \hfill & {{\text{if}}\,\gamma (i) = - 1.} \hfill \\ \end{array} } \right. $$

for $m_{i,j} \ne 0,\,i = 1, \ldots ,N,\,j = 1, \ldots ,N,k = 1, \ldots ,p(i) - 1$. A smooth nonlinear map is then obtained by fitting a polynomial smoothing spline.

## Numerical Simulation Example

The proposed algorithm is demonstrated using simulated data generated by the stochastic dynamical system

44 $$ x_{n + 1} = S(x_{n} ) + u_{n} + \xi_{n} \quad ( {\text{mod}}\, 1 ) , $$

where $S(x_{n} ) = 4x_{n} (1 - x_{n} ),\,S{:}\,[0,1] \to [0,1],\,u \in [0,1]$ is the input variable having the following density function that is truncated to the range of [0,1]

45 $$ f_{u} (u) = \frac{1}{2}\left( {\frac{1}{{\sigma_{1} \sqrt {2\pi } }}e^{{ - \frac{{(u - \mu_{1} )^{2} }}{{2\sigma_{1}^{2} }}}} + \frac{1}{{\sigma_{2} \sqrt {2\pi } }}e^{{ - \frac{{(u - \mu_{2} )^{2} }}{{2\sigma_{2}^{2} }}}} } \right), $$

$\mu_{1} = 0.30,\,\sigma_{1} = 0.70,\,\mu_{2} = 0.60,\,\sigma_{2} = 0.10$. The noise variable is assumed to have a non-Gaussian density function with compact support [− 0.2, 0.2] given by

46 $$ g(\xi ) = \left\{ {\begin{array}{*{20}l} {4,} \hfill &\quad - \,0.20 \le \xi \le - 0.10; \hfill \\ {{4 \mathord{\left/ {\vphantom {4 3}} \right. \kern-0pt} 3},} \hfill & \quad{ - \,0.10 < \xi \le 0.05;} \hfill \\ {{{20} \mathord{\left/ {\vphantom {{20} 7}} \right. \kern-0pt} 7},} \hfill &\quad {0.05 < \xi \le 0.12;} \hfill \\ {2.5,} \hfill &\quad {0.12 < \xi \le 0.20.} \hfill \\ \end{array} } \right. $$

**Fig. 1:**
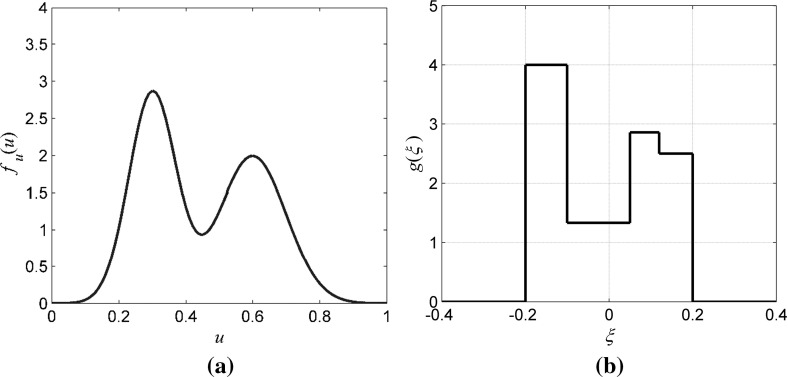
Probability density functions of the input *u* (**a**) and the noise *ξ* (**b**)

In practice, there are no restrictions on the shape of this density function. The density functions of the input and noise, $f_{u}$ and *g*, are shown in Fig. 1.

**Fig. 2:**
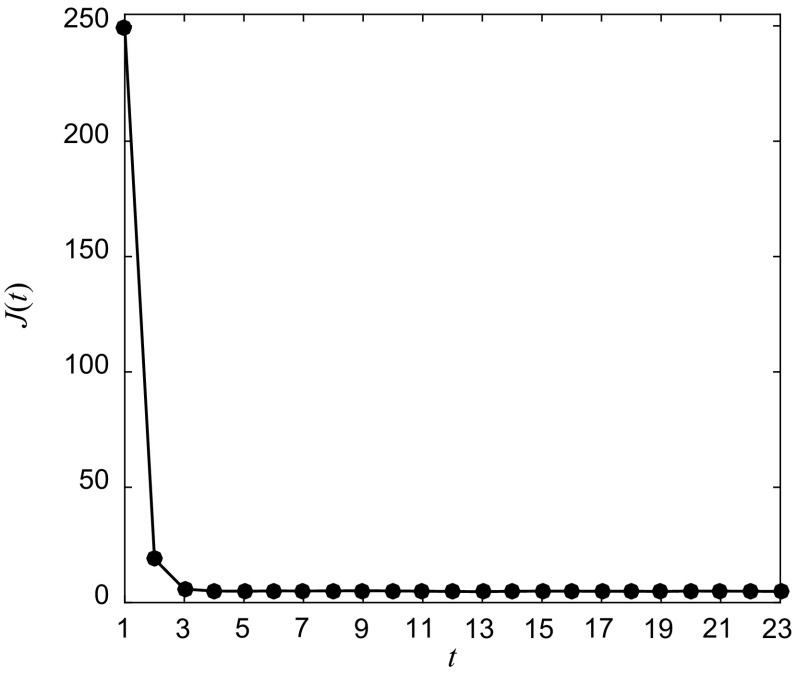
Results of the performance function *J*(*t*) in (32) for 1 ≤ *t* ≤ 23

For the purpose of inferring the piecewise linear semi-Markov transformation that approximates the original logistic map *S*, we define a uniform partition $\Re$ of [0, 1] with $N = 40$ intervals. To generate the data used in the reconstruction, $K = 40$ piecewise constant initial density functions $f_{0,i} = \chi_{{R_{i} }} (x),\,i = 1, \ldots ,40$ were sampled to generate the initial states $X_{0,i} = \{ x_{j}^{0,i} \}_{j = 1}^{{\theta_{1} }} ,\,\theta_{1} = 5 \times 10^{3} ,\,i = 1, \ldots ,40$. The input and noise densities were sampled to generate the input and noise data sets $U = \{ u_{i} \}_{i = 1}^{{\theta_{1} }}$ and $\varXi = \{ \xi_{i} \}_{i = 1}^{{\theta_{1} }}$. In total, 40 sequences of new states $X_{t,i} = \{ x_{j}^{t,i} \}_{j = 1}^{{\theta_{1} }} ,\,i = 1, \ldots ,40$ were then observed by iterating *t* times system (44) and these were subsequently used to estimate the corresponding piecewise constant densities $f_{t,i}^{N}$, $i = 1, \ldots ,40$, $t \ge 1$, over the uniform partition $\Re$. Figure 2 shows the calculated results of the performance function *J*(*t*) in (32), $1 \le t \le 23$, which represents the summing differences of each two successive densities of the *K* sequences of densities. As can be seen, the minimum can be found at $t \ge 4$, which suggests that densities in all the sequences approach the equilibrium distribution. It follows that $1 \le T \le 4$.

**Fig. 3:**
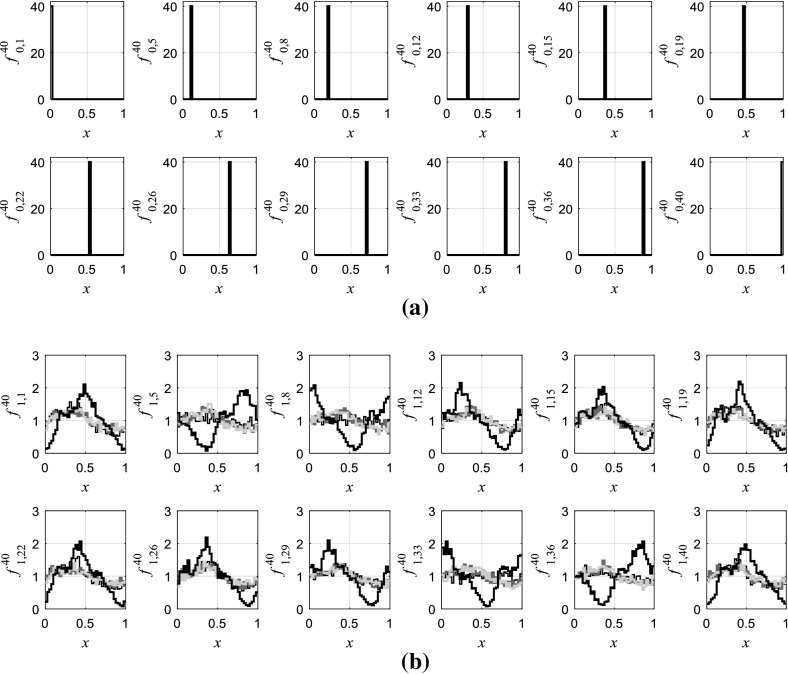
a Examples of initial densities and **b** the corresponding generated new densities at *T* = 1 represented by the black thick lines, densities at *T* = 2 represented by the black thin lines, densities at *T* = 3 represented by the dark gray lines and stationary densities generated after 1 × 10^4^ iterations that are represented by the light gray lines

Here we choose *T* = 1. Figure 3 shows the initial densities $f_{0,k}$ and their image densities $f_{1,k}^{N}$, which are used to reconstruct the approximate map, and also $f_{2,k}^{N}$, $f_{3,k}^{N}$ and the equilibrium observed after 1 × 10^4^ iterations. It can be seen that compared with $f_{1,k}^{N} ,\,f_{2,k}^{N}$ and $f_{3,k}^{N}$ are more close to the stationary density, and densities in each sequence rapidly converge to the same stationary density. This is also demonstrated in Fig. 2 that the derivative of $f_{i,k}$ at *i* = 3 is apparently lower than that of $f_{1,k}$ and $f_{2,k}$. Using the *lsqlin* function in the optimization toolbox of MATLAB to solve optimization problems (36), (38) and (40) in the proposed algorithm, the Frobenius–Perron matrix *M* is obtained.

**Fig. 4:**
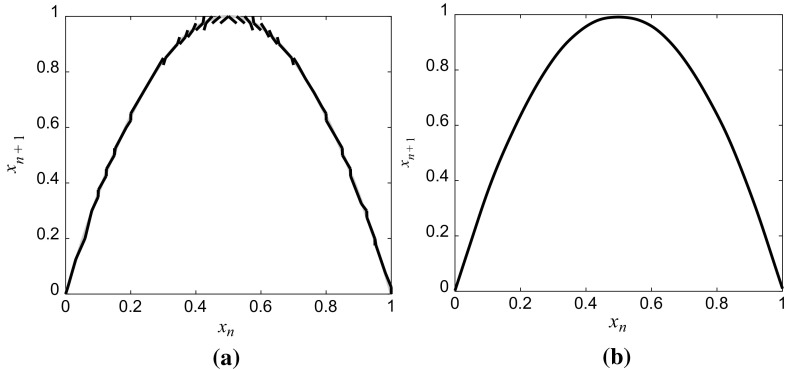
**a** The estimated piecewise linear semi-Markov transformation *Ŝ* and **b** the reconstructed continuous nonlinear map $\tilde{S}$

**Fig. 5:**
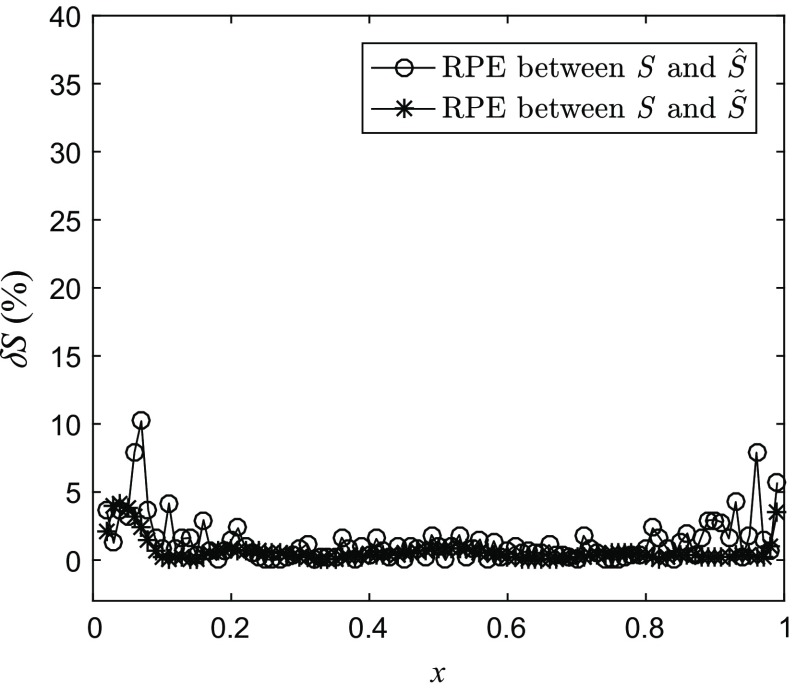
RPE between the original map *S* and the estimated piecewise linear semi-Markov transformation *Ŝ*, and also the reconstructed continuous nonlinear map $\tilde{S}$

The piecewise linear semi-Markov map $\hat{S}$ associated with the identified matrix *M* is shown in Fig. 4a. Finally, the continuous nonlinear map $\tilde{S}$ was estimated by fitting a cubic smoothing spline with the smoothing parameter 0.999, to a set of $10^{3}$ data points obtained by uniformly sampling the piecewise linear map $\hat{S}$ over [0, 1]. The reconstructed continuous nonlinear map is shown in Fig. 4b. The performance of the reconstruction algorithm is evaluated by computing the relative percentage error (RPE)

47 $$ \begin{aligned} & \delta {\kern 1pt} S(x|\hat{S}(x)) = 100\left| {\frac{{S(x) - \hat{S}(x)}}{S(x)}} \right|\,(\% ), \\ & \delta {\kern 1pt} S(x|\tilde{S}(x)) = 100\left| {\frac{{S(x) - \tilde{S}(x)}}{S(x)}} \right|\,(\% ), \\ \end{aligned} $$

between the original and estimated maps *Ŝ* and between the original and the smoothed map $\tilde{S}$, respectively, which is illustrated in Fig. 5. As can be seen, the full error for *Ŝ* and 95% of the error for $\tilde{S}$ is lower than 5%. With the increase of *N*, the estimated map *Ŝ* is more close to *S*.

**Fig. 6:**
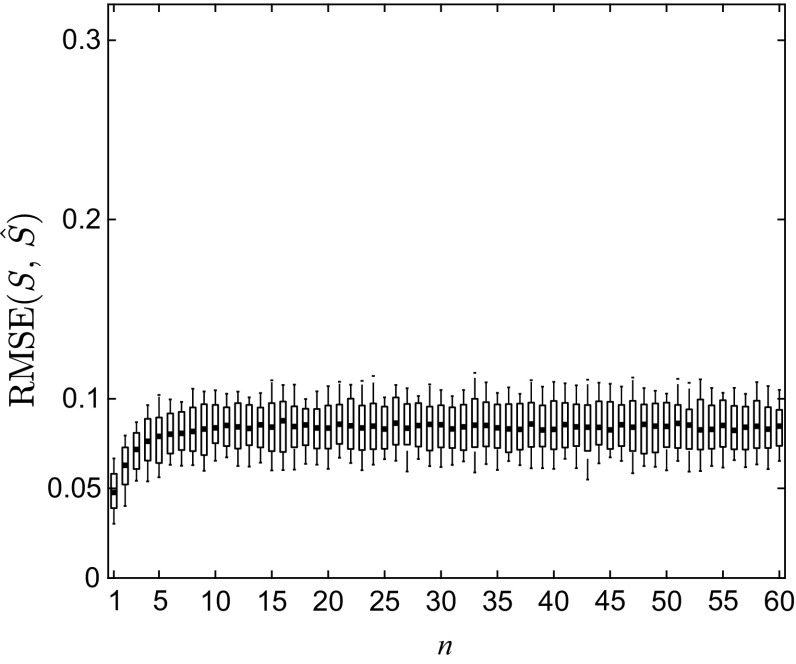
The mean, 10% and 90% quantiles of the 100 RMSE between $f_{n,k}^{40}$ and $\hat{f}_{n,k}^{40}$ for *k* = 1, …, 100 at *n* = 1, …, 60

**Fig. 7:**
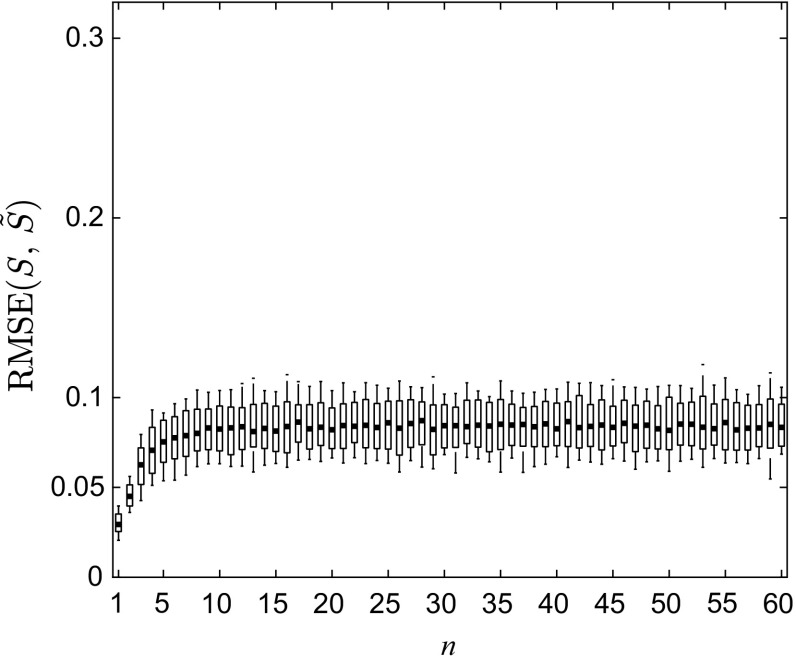
The mean, 10% and 90% quantiles of the 100 RMSE between $f_{n,k}^{40}$ and $\hat{f}_{n,k}^{40}$ at *n* = 1, …, 60

To further evaluate the accuracy of the reconstruction, the constructed piecewise linear semi-Markov approximation *Ŝ* and the estimated continuous map $\tilde{S}$ were used to predict *n*-iteration ahead density functions, $n = 1, \ldots ,60$, respectively, using a Gaussian distribution function ${\mathcal{N}}(0.6,\;0.4^{2} )$ truncated to [0, 1] as the initial state density function $f_{0}$, the input density given in (45) and the noise density given in (46). With 100 sets of $\theta_{2} = 1 \times 10^{4}$ input data $U = \{ u_{k,i} \}_{k = 1,i = 1}^{{100,\theta_{2} }}$ generated by sampling $f_{u}$ and 100 sets of the same number of noise data $\varXi = \{ \xi_{k,i} \}_{k = 1,i = 1}^{{100,\theta_{2} }}$ from *g*, 100 sets of *θ*_2_ randomly distributed initial states $X_{0,k} = \{ x_{j}^{0,k} \}_{j = 1}^{{\theta_{2} }}$, *k* = 1, …, 100, were separately iterated for 60 steps using stochastic model (1) with the original map *S*, the identified piecewise linear semi-Markov approximation *Ŝ* and the estimated continuous map $\tilde{S}$, respectively. In each step piecewise constant density functions $f_{n,k}^{40} (x) = \sum\nolimits_{j = 1}^{40} {w_{j}^{n,k} \chi_{{R_{j} }} (x)} ,\,\hat{f}_{n,k}^{40} (x) = \sum\nolimits_{j = 1}^{40} {\hat{w}_{j}^{n,k} \chi_{{R_{j} }} (x)} ,$ and $\tilde{f}_{n,k}^{40} (x) = \sum\nolimits_{j = 1}^{40} {\tilde{w}_{j}^{n,k} \chi_{{R_{j} }} (x)}$, *k* = 1, …, 100, *n* = 1, …, 60, are then estimated over $\Re$ from the generated states. The root-mean-square error (RMSE) between $f_{n,k}^{40}$ and $\hat{f}_{n,k}^{40}$ and between $f_{n,k}^{40}$ and $\tilde{f}_{n,k}^{40}$ is calculated by

48 $$ \begin{aligned} {\text{RMSE}}(S,\hat{S})^{n,k} & = \sqrt {\frac{1}{40}\sum\limits_{i = 1}^{40} {(w_{i}^{n,k} - \hat{w}_{i}^{n,k} )^{2} } } , \\ {\text{RMSE}}(S,\tilde{S})^{n,k} & = \sqrt {\frac{1}{40}\sum\limits_{i = 1}^{40} {(w_{i}^{n,k} - \tilde{w}_{i}^{n,k} )^{2} } } , \\ \end{aligned} $$

where $\hat{w}_{i}^{n,k}$ and $\tilde{w}_{i}^{n,k}$ are the coefficients of predicted density function using *Ŝ* and $\tilde{S}$, respectively. The mean, 10 and 90% quantiles of the 100 RMSEs for *Ŝ* and $\tilde{S}$ at each iteration are shown in Figs. 6 and 7. As can be seen from them, 90% quantiles of the error remain constantly less than 0.1 for both *Ŝ* and $\tilde{S}$, and their mean values stabilize around 0.08 after 10 iterations.

**Fig. 8:**
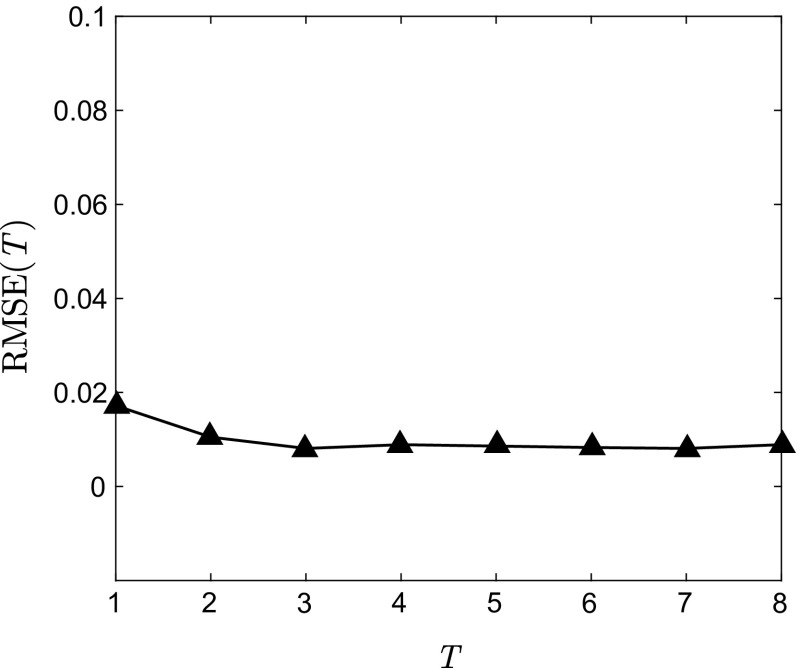
RMSE between *S* and *Ŝ* on 100 uniformly spaced points in [0, 1] for *T* = 1, …, 8

Figure 8 shows the RMSE between *S* and *Ŝ* on 100 uniformly spaced points within $\Re$ for *T* = 1, …, 8. As can be seen, a small decrease of the error occurs from *T* = 1 till 4, and then the error maintains almost constant for $T \ge 4$. This implies that all the sequences reach the equilibrium distribution after 4 iterations, which is in consistent with Fig. 2. From Fig. 2, distance between $f_{1,k}$ and $f_{*}$ is remarkably larger than that between $f_{n,k}$ and $f_{*}$ for *n* = 2,3,4, and therefore the error is slightly diminished even though more new densities are added for the identification.

## Conclusions

This paper introduced a new algorithm for reconstructing the underlying one-dimensional map for an autonomous dynamical system that is driven by an additive control input and also subjected to an additive stochastic perturbation, given the observed sequences of probability density functions generated by the unknown system, and the input and noise density functions. Evolution of densities was formulated and described by a stochastic Frobenius–Perron operator that has a matrix representation. This forms the basis for the algorithm to identify the Frobenius–Perron matrix associated with a piecewise linear semi-Markov approximation to the underlying nonlinear map. Based on the matrix representation of the stochastic Frobenius–Perron operator the densities generated by the dynamical system and evolving from a given initial condition can be predicted. Convergence of the evolving densities analyzed from the matrix representation reveals a fact that only a limited number of densities characterizing the transient dynamics is observable for arbitrary initial condition, and thus, this requires different initial conditions so as to generate as many as possible temporal sequences of densities to reconstruct the underlying map.

For the situations where only a limited number of initial conditions are available for generating the temporal sequences of densities that converges quickly to the equilibrium distribution, a potential effective solution is to apply multiple linearly independent input density functions to the stochastic dynamical systems so that the densities would diverge to different equilibrium distributions, which will be further explored. From a practical perspective, it is also worthwhile to extend the approach to higher-dimensional systems based on sequences of mixture densities generated by the more complex systems.

Furthermore, this paper provides a new insight into identification of stochastic dynamical systems given the density functions of control inputs. It triggers a new scheme to solve the control problem for such systems. Specifically, given the noise density function, the problem aims to determine the optimal input density function so that the dynamical system can have a desired equilibrium distribution that represents the targeted asymptotic dynamics.
